# Robust estimation of time-dependent precision matrix with application to the cryptocurrency market

**DOI:** 10.1186/s40854-022-00355-4

**Published:** 2022-05-05

**Authors:** Paola Stolfi, Mauro Bernardi, Davide Vergni

**Affiliations:** 1 Institute for Applied Mathematics “Mauro Picone” (IAC) - National Research Council of Italy, Via dei Taurini 19, 00185 Rome, Italy; 2 Department of Statistical Sciences, University of Padova, Via C. Battisti, 241, 35121 Padua, Italy

**Keywords:** Time-varying models, Robust methods, Kernel estimation, Precision matrix, Divergence

## Abstract

Most financial signals show time dependency that, combined with noisy and extreme events, poses serious problems in the parameter estimations of statistical models. Moreover, when addressing asset pricing, portfolio selection, and investment strategies, accurate estimates of the relationship among assets are as necessary as are delicate in a time-dependent context. In this regard, fundamental tools that increasingly attract research interests are precision matrix and graphical models, which are able to obtain insights into the joint evolution of financial quantities. In this paper, we present a robust divergence estimator for a time-varying precision matrix that can manage both the extreme events and time-dependency that affect financial time series. Furthermore, we provide an algorithm to handle parameter estimations that uses the “maximization–minimization” approach. We apply the methodology to synthetic data to test its performances. Then, we consider the cryptocurrency market as a real data application, given its remarkable suitability for the proposed method because of its volatile and unregulated nature.

## Introduction

Most financial signals show time dependency, which, when combined with the presence of noisy and extreme events, can cause severe difficulties in the estimation of statistical models, particularly for the asset allocation problem in which relationships among financial quantities play a fundamental role. In this work, we model the joint dynamics of financial signals, handling both the time-dependency of the time series and the presence of extreme events—two features characterizing most financial time series and, in particular, the cryptocurrency (CC) market. The first CC—Bitcoin—was developed in 2009. At the end of 2015, the number of available CCs was approximately five Hundred. In mid-2021, more than six thousand CCs existed. These numbers, together with the growing literature regarding these new currencies, are witnesses to how the interest in CCs increased during recent years. Several statistical aspects related to CCs were studied, as in Bariviera et al. (2017), Cunha and Da Silva (2020), Cont (2001) and Hu et al. (2019), which analyzed stylized characteristics of the CC market. Alternatively, in Ciaian and Rajcaniova (2018) and Brandvold et al. (2015), the authors investigated the joint dynamics of CCs. Moreover, several authors investigated CCs’ volatility using GARCH models and their extensions, including Caporale and Zekokh (2019), Dyhrberg (2016), and Katsiampa (2017). Robustness issues regarding GARCH modeling of CCs’ volatility were addressed by Charles and Darné (2019). Beyond multivariate GARCH models, Bouri et al. (2017) used a dynamic conditional correlation model to describe the time-varying conditional correlation between Bitcoin and other financial assets, whereas Corbet et al. (2018) studied the time-varying relationships between some CCs and other financial assets using a pairwise directional measure of volatility spillovers. In Bazán-Palomino (2020, 2021), the authors investigated the correlations between Bitcoin and its forks, where the terms fork refers to a new version of the blockchain obtained by introducing changes in the rules of the original blockchain. The authors used several volatility models—both univariate and bivariate—and showed that Bitcoin and their forks are dynamically correlated. Chaim and Laurini (2019) modeled the returns and volatility dynamics of CCs using a multivariate stochastic volatility model with discontinuous jumps, whereas Fry (2018) developed bubble models for CCs to handle the presence of heavy tails. To conclude this overview of CCs’ modeling literature, it is worth citing Catania et al. (2019), who proposed several models to improve CCs’ forecasting performances.

Notwithstanding the large number of potential applications of CCs, such as near real-time micropayments that are necessary to the development of the Internet of Things, currently, they are mainly used as speculative investment instruments. Indeed, many online exchange websites offer the opportunity to sell and buy all available CCs and to create investment portfolios to manage the related financial risk. However, given their nature, these currencies are far from being similar to the traditional ones (Baek and Elbeck 2015; Huang et al. 2019)—an aspect that immediately emerges by inspecting the data. Several researchers analyzed the opportunity to invest in CCs: Briere et al. (2015) analyzed the returns of traditional asset portfolios in which bitcoin was inserted. Klein et al. (2018) investigated the performance of a portfolio containing both traditional assets and several CCs and tested the robustness of their results considering the CC index *CRIX*. Chuen et al. (2017) analyzed the investing opportunities of CCs by studying the top ten CCs together with the *CRIX* index and other indexes related to traditional assets. In all of these cases, of paramount importance is to estimate the degree of dependency between the various CCs.

In this work, we study the dependencies among many CCs in an attempt to provide an instrument to investigate the behavior of the entire cryptocurrency market. To this end, we study the precision matrix, namely, the inverse of the correlation matrix, whose elements—when dealing with Gaussian distributions—represent the dependence between two variables conditionally on the remaining ones (Lauritzen 1996); that is, if the element *i*, *j* of the precision matrix is zero, then the variables *i* and *j* of the multivariate distribution are conditionally independent. We do not study the correlation matrix because it may not provide meaningful information in multivariate settings on the dependence between the variables. Indeed, two variables might show significant correlation because of the influence of other variables. Models based on the precision matrix are called *graphical models*, which means that the precision matrix allows for a graph to be built whose nodes represent the variables—the considered financial time series—and the edges represent the conditional dependence; see Lauritzen (1996) for more details. In high-dimensional settings, the opportunity to build a graph is worthwhile because it allows for interactions to be visualized, clusters to be created, and in general, the exploitation of the entire apparatus of graph theory, which can reveal the hidden topological properties of the financial network. These models are frequently used in the statistical and machine learning literature for certain reasons. However, despite their theoretical relevance, their practical applicability is limited by the restrictive assumptions of independent and identically distributed (i.i.d.) Gaussian observations that are often principally unrealistic because of the presence of a non-Gaussian or a contaminated Gaussian process—or the time-dependent nature of the process. The body of literature contains studies addressing the distortion arising from a Gaussian assumption in the case of heavy tails: Lafferty et al. (2012) proposed two non-parametric approaches, one based on a transformation of the data and the second based on a kernel density estimation; Finegold and Drton (2014) proposed in a Bayesian setting Dirichlet t-distributions to handle heavy tails and investigated the computational costs derived from the Gibbs sampler algorithm tailored for such distributions; Vogel and Tyler (2014) proposed graphical M-estimators tailored for elliptical graphical models; and Hirose et al. (2017) proposed a robust estimator that minimizes the $\gamma$-divergence between the observed and theoretical distribution. Moving on the non-stationary issue, Zhou et al. (2010) investigated time-dependent graphical models by using a temporal kernel to address a precision matrix whose structure evolves smoothly over time. To the best of our knowledge, no studies simultaneously handled extreme events and time-dependency: our goal in this work is to fill this gap by developing an estimation method for multivariate time series that handle time-dependent parameters and that is robust against the presence of extreme events. In particular, we propose a robustification based on $\gamma$-divergence of the time-varying approach introduced in Zhou et al. (2010). Using a kernel estimator together with an M-type estimator was introduced by Cai and Ould-Saïd (2003), who proposed a robust version of a local linear regression smoother for stationary time series. They also extended the asymptotic theory developed by Fan and Jiang (2000) to the case of non-i.i.d. random variables, in which the authors studied local robust techniques in the i.i.d. case. Our approach is similar to that proposed in Cai and Ould-Saïd (2003) because it merges an M-type estimator with a kernel estimator but differs in the model considered.

The remainder of this paper is organized as follows. “Model” section describes the statistical model considered in this work. “Estimation methodology” section addresses the methodological contribution of the paper and introduces details regarding time-varying and robust estimations through $\gamma$-divergences that are the starting points of our estimator. “Test case” section addresses simulation experiments constructed to test the performance of the proposed methodology. “Cryptocurrency market application” section describes the real data CCs’ application, and “Discussion” and “Conclusions” sections provide discussions and the conclusions, respectively.

## Model

The simplest model accounting for financial price dynamics is given by the geometric Brownian motion $\mathrm {d}p_t=\mu p_t\mathrm {d}t+\sigma p_t\mathrm {d}W_t$, where $\mu$ and $\sigma$ are the percentages of the drift and volatility, respectively, and $\mathrm {d}W_t$ is a simple Brownian motion with independent increments normally distributed. The logarithm of the price follows a simple Brownian motion. As is usually done, we work with the log return of the price, $r_{t}=\text{ log }\frac{p_{t}}{p_{t-1}}$.

We assume that return dynamics are described by a contaminated time-dependent Gaussian process. Formally, we assume that the observed log returns $\varvec{r}_{t}$ are distributed according to the following probability: distribution

1 $$\begin{aligned} g\left( \varvec{r}_t, t\right) = \left( 1-\epsilon \right) f\left( \varvec{r}_t, t\right) +\epsilon s\left( \varvec{r}_t, t\right) \end{aligned}$$

where $f\left( \varvec{r}_t, t\right) = \mathsf {N}_{d}\left( \varvec{0}, \varvec{\varTheta }^{-1}_t\right)$ is a zero mean multivariate Gaussian distribution with a time-dependent precision matrix $\varvec{\varTheta }_t$ (that is, the inverse of the correlation matrix), $s\left( \varvec{r}_t, t\right)$ is a contaminating probability distribution accounting for the shock components, and $\epsilon$ is the ratio of contamination that will never be considered as infinitesimal—meaning that we are interested in the case of a significant contamination of extreme events. Worth noting is that Eq. (1) is just a mathematical formulation to describe a contaminated model in which, in addition to the selected statistical process, extreme events, outliers, or some other possible events are present that are not described by the model $f(\cdot )$. Therefore, the contamination ratio $\epsilon$ should not be considered a model parameter to be estimated, and the only parameters of interest are those of $f\left( \varvec{r}_t, t\right) = \mathsf {N}_{d}\left( \varvec{0}, \varvec{\varTheta }^{-1}_t\right)$.

Moreover, the distribution $f\left( \varvec{r}_t, t\right)$, which is assumed to be Gaussian to keep the presentation of the method as clear as possible, can be replaced by a general conditional probability distribution with Gaussian noise, leaving the formalism unchanged.

The choice of this model is motivated by the interest in the conditional correlations among CCs, which are represented by the elements of the precision matrix when addressing Gaussian distributions.

## Estimation methodology

This section describes the main contribution of this study, namely, the presentation of a local robust estimator of the precision matrix described in “Robust time-varying estimation” section. The proposed estimator is constructed using two estimators: the local estimator of the time-varying precision matrix introduced in Zhou et al. (2010) and briefly recalled in “Time-varying estimation” section, and the robust estimator based on $\gamma$-divergence introduced in Fujisawa and Eguchi (2008) and briefly recalled in “Robust estimation” section.

### Time-varying estimation

In Zhou et al. (2010), the authors are interested in the local estimation of the precision matrix of the model $f\left( \varvec{r}, t\right) = \mathsf {N}_{d}\left( \varvec{0}, \varvec{\varTheta }^{-1}_t\right)$. To this end, they proposed a kernel estimator defined as follows.

Let $\left\{ \varvec{r}_{t}\right\} _{t=1}^{T}$ be a sample of T observations of $\varvec{r}_{t}$, as previously defined. The time-varying precision matrix $\varvec{\varTheta }_{t}$ in the non-i.i.d. case is estimated using the modified Lasso-penalized maximum likelihood estimator obtained by introducing a kernel over the time domain,

2 $$\begin{aligned} \widehat{\varvec{\varTheta }}\left( t\right) = \arg \min _{\varvec{\varTheta }\succ 0}\left\{ \text{ tr }\left( \varvec{\varTheta }\widehat{\varvec{S}}\left( t\right) \right) -\ln \vert \varvec{\varTheta }\vert +\lambda \Vert \varvec{\varTheta }\Vert _{1}\right\} , \end{aligned}$$

where $\left( \widehat{\varvec{S}}\left( t\right) \right) _{i,j} = \sum _{u=1}^{T}w_{ut}\varvec{r}_{i,u}\varvec{r}_{j,u}^{\prime }$, is an empirical covariance matrix weighted with a symmetric time-dependent nonnegative kernel $w_{ut}=\frac{K\left( \vert u-t\vert /h\right) }{\sum _{u=1}^{T}K\left( \vert u-t\vert /h\right) }$ where $K\!(\cdot )$ is a Gaussian kernel, *h* is the bandwidth, and $\lambda \Vert \varvec{\varTheta }\Vert _{1}$ is the Lasso penalty added to local likelihood of inducing sparsity in the precision matrix. Noteworthy is that the bandwidth parameter *h* acts on the kernel by modifying its shape; that is, it is the standard deviation of the Gaussian kernel. In particular, low values of *h* provide high weights only to points close to the reference time *t*, whereas high values of *h* translate into assigning relevant weights to points far from the reference time *t*. In other words, the bandwidth parameter *h* determines the estimation window size.

The estimator defined in Eq. (2) has good asymptotic properties if the precision matrix evolves smoothly over time. At varying *t*, it provides a chain of graphs that unveils how the multivariate structure of the process evolves over time—exactly the objective of this study. Unfortunately, the maximum-likelihood approach suffers because of its non-robust nature (Huber and Ronchetti 2011), the presence of contamination from extreme random events. This issue is addressed in the next subsection.

### Robust estimation

Many robust approaches are developed in the statistical literature. Some are based on the modification of the minimization function to obtain a finite influence function [see Huber and Ronchetti (2011)]; others are based on modifying the Kullbach–Leibler divergence, whose minimization corresponds to the maximum-likelihood approach to obtain a divergence that provides less weight to extreme events or outliers; thus, it is robust against them [see Basu et al. (1998) and Fujisawa and Eguchi (2008)]. In this study, we followed the divergence-based approach; in particular, we consider the one introduced in Fujisawa and Eguchi (2008) based on $\gamma$-divergence. To introduce this methodology, we use the model defined in Eq. (1): in the i.i.d case, namely,

$$\begin{aligned} g\left( \varvec{r}\right) = \left( 1-\epsilon \right) f\left( \varvec{r}\right) +\epsilon s\left( \varvec{r}\right) . \end{aligned}$$

Now, let $\left\{ \varvec{r}_{t}\right\} _{t=1}^T$ be an i.i.d. sample drawn from *g*. In this framework, a non-robust approach chooses the estimated parameters $\hat{\varvec{\theta }}$ such that $f_{\hat{\varvec{\theta }}}$ is close to *g* and, thus, is biased by outliers. Instead, a robust approach chooses the estimated parameters $\hat{\varvec{\theta }}$ such that $f_{\hat{\varvec{\theta }}}$ is close to *f*. The choice of the divergence depends on the specific problem. In this paper, we consider the $\gamma$-divergence defined as

3 $$\begin{aligned} d_{\gamma }\left( g,f\right) = -\frac{1}{\gamma }\text{ log }\int g\left( \varvec{r}\right) f\left( \varvec{r}\right) ^{\gamma }\mathrm {d}\varvec{r}+\frac{1}{1+\gamma }\text{ log }\int f\left( \varvec{r}\right) ^{1+\gamma }\mathrm {d}\varvec{r}, \end{aligned}$$

where $\gamma >0$ is constant. Clearly, $f\left( \varvec{r}\right) ^{\gamma }$ weights the observations—in particular, small values of $\gamma$ emphasized extreme events, whereas large values of $\gamma$ emphasized the underlying distribution. The second term in Eq. (3) is needed to have an unbiased estimator. The $\gamma$-divergence is empirically estimated by

4 $$\begin{aligned} d_{\gamma }\left( \bar{g}, f_{\varvec{\theta }}\right)&=-\frac{1}{\gamma }\ln \left\{ \frac{1}{T}\sum _{t=1}^{T}f\left( \varvec{r}_{t}\right) ^{\gamma }\right\} +\frac{1}{1+\gamma }\ln \int f\left( \varvec{r}\right) ^{1+\gamma }\mathrm {d}\varvec{r} \end{aligned}$$

5 $$\begin{aligned}&=\ell _{1}\left( \varvec{r}_{t},\varvec{\theta }\right) +\ell _{2}\left( \varvec{\theta }\right) , \end{aligned}$$

Thus, the robust estimator is obtained as solution to the minimization problem defined as

$$\begin{aligned} \widehat{\varvec{\theta }}=\arg \min _{\varvec{\theta }}d_{\gamma }\left( \bar{g}, f_{\varvec{\theta }}\right) , \end{aligned}$$

where we changed the notation from *f* to $f_{\varvec{\theta }}$ to highlight the dependence over the set of parameters $\varvec{\theta }$ and this estimator exhibited good asymptotic properties based on the theory of *M*estimators (DasGupta 2008) and shows a small bias even in the case of heavy contamination assuming that $f\left( \varvec{r}\right)$ is sufficiently small when $\varvec{r}$ is an outlier; that is, the following condition must be respected

$$\begin{aligned} \left\{ \int s\left( \varvec{r}\right) f\left( \varvec{r}\right) ^{\gamma } \mathrm {d}\varvec{r} \right\} ^{\frac{1}{\gamma }} \qquad \text{ is } \text{ small } \text{ for } \text{ a } \text{ sufficiently } \text{ large } \gamma >0\text{. } \end{aligned}$$

Usually, $\gamma < 1$ is considered; for further details see Fujisawa and Eguchi (2008). However, this estimator does not handle the time-varying nature of the problem considered.

### Robust time-varying estimation

In this section, we present the main methodological contribution of this paper. That is, we propose an estimator that can manage the time-varying nature of the problem and the presence of extreme events contaminating the underlying distribution. To this end, we modify the estimated $\gamma$-divergence defined in Eq. (4) to assign greater importance to observations close to the period considered. We apply the same methodology as in Zhou et al. (2010) that introduces a kernel over the time domain, as detailed in “Time-varying estimation” section. Here, we consider the complete model defined in Eq. (1), which we report here for the reader’s convenience

$$\begin{aligned} g\left( \varvec{r}_t, t\right) = \left( 1-\epsilon \right) f\left( \varvec{r}_t, t\right) +\epsilon s\left( \varvec{r}_t, t\right) . \end{aligned}$$

Using the same notation introduced in the previous sections, we define the local estimated $\gamma$-divergence as

6 $$\begin{aligned} d\left( \bar{g}, f_{\varvec{\theta }_{t}}\right)&= -\frac{1}{\gamma }\ln \left\{ \sum _{u=1}^{T}w_{ut}f\left( \varvec{r}_{u}, \varvec{\theta }_{t}\right) ^{\gamma }\right\} +\frac{1}{1+\gamma }\ln \int f\left( \varvec{r},\varvec{\theta }_{t}\right) ^{1+\gamma }\mathrm {d}\varvec{r}\nonumber \\&=\ell _{1, t}\left( \varvec{r}_{t},\varvec{\theta }_{t}\right) +\ell _{2}\left( \varvec{\theta }_{t}\right) . \end{aligned}$$

This estimator considers both extreme events through the parameter $\gamma$ and the time dependency through the kernel $w_{ut}=\frac{K\left( \vert u-t\vert /h\right) }{\sum _{u=1}^{T}K\left( \vert u-t\vert /h\right) }$ with bandwidth *h*. Indeed, the parameter $\gamma$ weighs observations according to the underlying distribution, as detailed in “Robust estimation” section, whereas the kernel $w_{ut}$ provides significant weight only to observations at a temporal distance less than the bandwidth *h* from the reference time *t*.

Thus, the local robust estimator at time *t* for $0\le t\le T$ is obtained as a solution to the following minimization problem:

7 $$\begin{aligned} \widehat{\varvec{\theta }}\left( t\right) =\arg \min _{\varvec{\theta }}d\left( \bar{g}, f_{\varvec{\theta }_{t}}\right) . \end{aligned}$$

The minimization problem defined in Eq. (7) can be solved through a maximization–minimization (MM) algorithm following the procedure detailed in Hirose et al. (2017). We customize our estimator in “Appendix 2”.

In high-dimensional settings—when the number of features is higher than the number of observations—sparsity can be introduced into the precision matrix structure by adding a penalty to the local estimated $\gamma$-divergence in Eq. (6).

Worth noting is that the use of a kernel estimator with an M-type estimator was introduced by Cai and Ould-Saïd (2003), and the authors proposed a robust version of a local linear regression smoother for stationary time series. They also extended the asymptotic theory developed by Fan and Jiang (2000) to the case of non-i.i.d. random variables, for which the authors studied local robust techniques in the i.i.d. case. We are working on adapting the asymptotic theory developed in Cai and Ould-Saïd (2003) to our estimator; however, the technicalities and mathematical proofs, which are beyond the scope of this work, will be published in a separate paper.

## Test case

In this section, we investigate the performance of the proposed estimator using a synthetic stochastic model with known parameters. In particular, in a time interval $t\in \left[ 0:T\right]$, we consider a distribution $g\left( \varvec{r}, t\right)$ obtained as a mixture of an underlying distribution $f\left( \varvec{r}, t\right)$ given by a 2-dimensional zero-mean time-dependent Gaussian random process and a contaminating distribution, $s\left( \varvec{r}\right)$, given by a simple two-dimensional Gaussian process with a large mean mimicking extreme events, as follows:

$$\begin{aligned} g\left( \varvec{r}, t\right) = \left( 1-\epsilon \right) f\left( \varvec{r}, t\right) + \epsilon s\left( \varvec{r}\right) = \left( 1-\epsilon \right) \mathcal {N}_{2}\left( \varvec{0}, \varvec{\varSigma }_{t}\right) + \epsilon \mathcal {N}_{2}\left( 10*\varvec{1}, \varvec{D}\right) . \end{aligned}$$

The covariance matrices $\varvec{\varSigma }_{t}$ and $\varvec{D}$ are defined as

$$\begin{aligned} \varvec{\varSigma }_{t}=\left[ \begin{matrix} \sigma ^2_{1} &{} \sigma _1\sigma _2\rho _{t}\\ \sigma _1\sigma _2\rho _{t} &{} \sigma ^2_{2} \end{matrix}\right] \quad \text{ and }\quad \varvec{D}=\left[ \begin{matrix} \sigma _{1}^{2} &{}0\\ 0 &{} \sigma _{2}^{2} \end{matrix}\right] , \end{aligned}$$

where $\sigma _{1}^{2} =0.197$ and $\sigma _{2}^{2} =0.363$ are taken as the sample variances of the Bitcoin and Ethereum time series, respectively, detailed in “Data” section. We consider various contamination rates $\epsilon$, and the time dependency in the underlying process is the result of a correlation coefficient $\rho _t$, which varies in $\left( -1, 1\right)$ in the considered time interval $\left[ 0:T\right]$, following the sigmoid function

$$\begin{aligned} \rho _{t}=\frac{\frac{t-t_0}{a}}{\sqrt{1+\left( \frac{t-t_0}{a}\right) ^2}}, \end{aligned}$$

where $$t_0=T/2$$ and $$a=T/10$$.

**Fig. 1:**
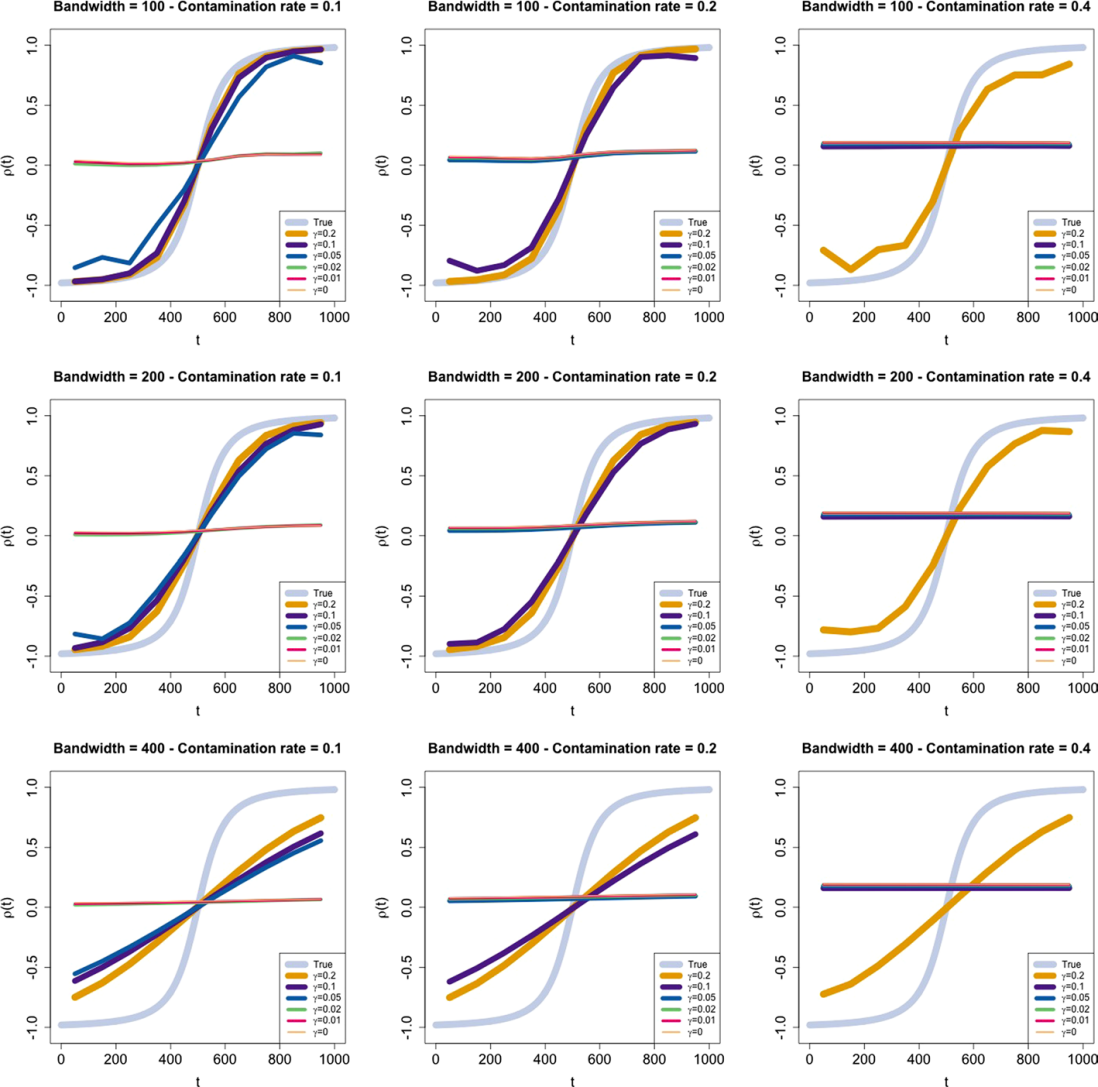
Dynamics of the estimated correlation coefficient. The parameters are estimated at 10 points, that is, $t^*=50, 150, 250, \ldots , 950$. Each row corresponds to a bandwidth, that is, $h=100, 200$, and 400, whereas each column corresponds to a contamination rate, that is, $\epsilon =0.1, 0.2$, and 0.4

At fixed time $t^*=50, 150, 250, \dots , 950$, following the algorithm of “Data” section, the values of the estimated correlation $\hat{\rho }\left( t^*\right)$ associated with different values of the contamination rate $\epsilon =0.1, 0.2, \text{ and }\ 0.4$ and obtained for several robustness parameter values $\gamma =0, 0.01, 0.02, 0.05, 0.1, \text{ and }\ 0.2$ and several bandwidth values $h=100, 200, \text{ and }\ 400$ are shown in Fig. 1. In each row of the figure, the bandwidth is fixed, whereas the contamination rate in each column is fixed, as labeled in the title of each panel. The results show that the performance of the estimator changes according to both the contamination rate and the $\gamma$ parameter. Specifically, for low values of $\gamma$ ($\gamma =0, 0.01, 0.02$), the estimate $\hat{\rho }\left( t^{*}\right)$ is not able to catch the dynamic of the correlation coefficient, $\rho _{t}$, of the underlying distribution, $f\left( \varvec{r}, t\right)$, as expected. For values greater than $\gamma =0.05$, the robust estimator privileged the underlying distribution, and the estimate $\hat{\rho }\left( t^{*}\right)$ identifies the true correlation dynamics. Clearly, increasing the contamination rate requires an increase in the value of $\gamma$ to obtain a good estimate of $\rho _{t}$ . Worth noting is that the values of $\gamma$ chosen in these simulations point out (see the first row of Fig. 1) that when the contamination is small, higher values of $\gamma$ do not improve the estimate, whereas higher values of $\gamma$ are needed to improve the estimate. We could have chosen more values of $\gamma$; however, to understand the method and the readability of the related plots, we found that the proposed values were a good choice. Bandwidth *h* acts as a smoothing parameter and softens the variation in the true correlation dynamics. Truly remarkable is that a standard non-robust estimator (the case of small $\gamma$) is absolutely unable to grasp the underlying process—even in this simple example—and is completely diverted from the contamination of extreme events.

## Cryptocurrency market application

Recently, the precision matrix has attracted increasing interest in the financial context, given its various and useful applications, from portfolio optimization, in which the closed-form solution to the global minimum variance portfolio involves the precision matrix (Torri et al. 2019), to systemic risk analysis, in which the partial correlations are more informative than independent correlations when analyzing the financial system as a whole. Many measures based on the precision matrix have been proposed to assess both systemic risk and portfolio strategy performances; see, for instance, Senneret et al. (2016) and Torri et al. (2019).

In this real data application, we show how the estimation of CCs’ precision matrix (together with other interesting financial quantities) changes according to the choice of the estimator, that is, when considering a robust approach against a non-robust one or assuming a time-varying approach instead of a time-invariant one. The existence and extent of these variations must necessarily be considered by those decision makers who rely on financial quantity estimates associated with different time horizons.

### Data

For this study, we consider the daily close value, $p_{t}$, of 12 CCs, namely, Bitcoin (BTC), Ethereum (ETH), XRP (XRP), Litecoin (LTC), Stellar (XLM), Monero (XMR), Dash (DASH), Ethereum Classic (ETC), NEM (XEM), Zcash (ZEC), Dogecoin (DOGE), and Waves (WAVES), from 01/02/2017 to 26/07/2021, downloaded from CoinMarketCap (https://coinmarketcap.com/it/). The dynamics of CCs’ log returns and histograms (Figs. 3, 4) make it evident that their distribution cannot be considered Gaussian because of the presence of extreme events. Such a feature is confirmed by the sample statistics in Table 1, in which we report robust statistics, namely median, interquartile range, measures of skewness, and excess kurtosis based on quantiles [see Kim and White (2004)], minimum, and maximum. We also performed the Jarque-Bera test for non-normality but did not include the p-values, all of which were zero. Some CCs, such as *XRP*, *XEM*, and *DOGE*, exhibit very high kurtosis, confirming the presence of extreme events.

**Fig. 2:**
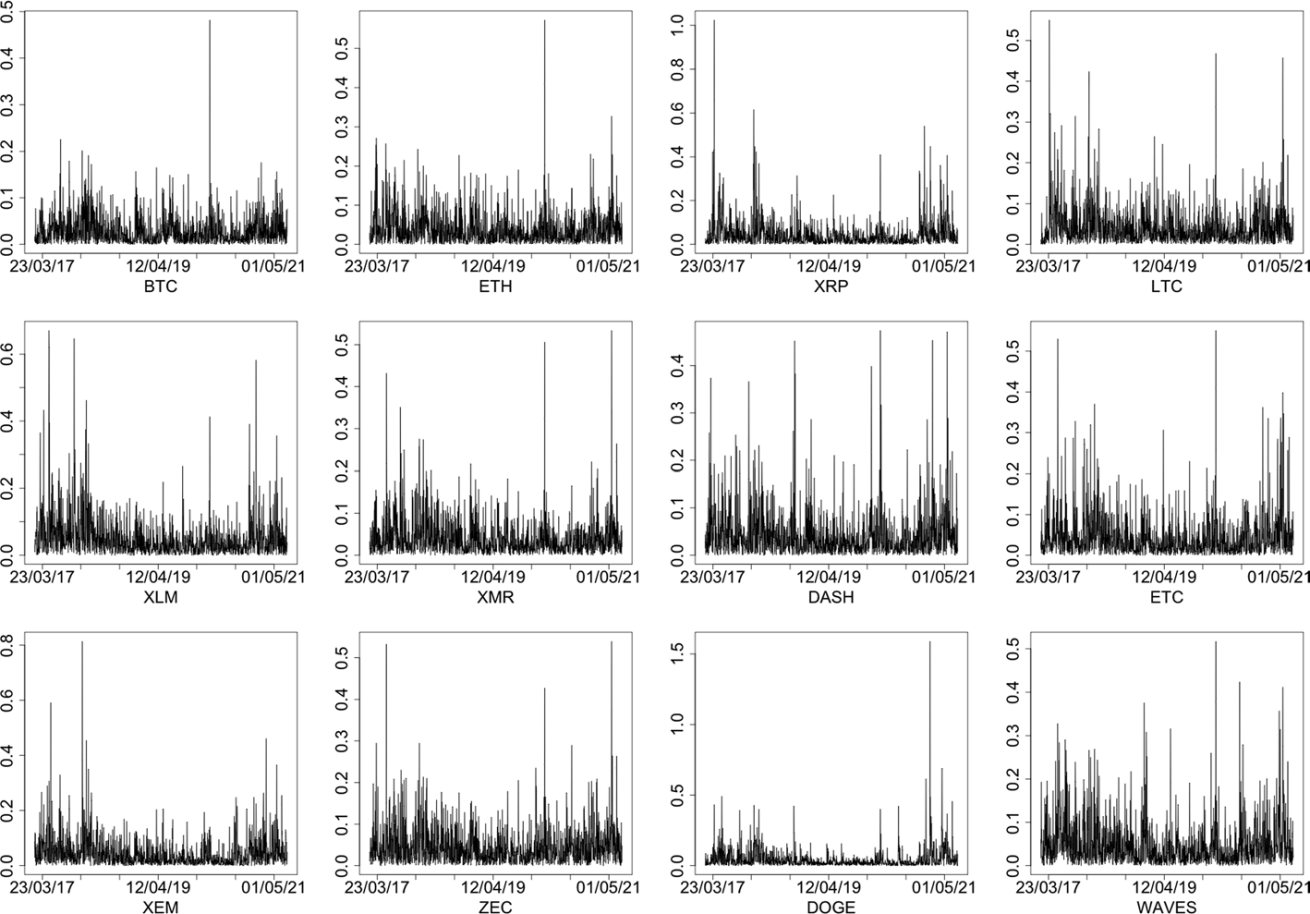
Dynamics of $\vert r_{t}-\mu \vert$, where $r_{t}$ are the 12 CCs’ log return considered and $\mu$ is the corresponding mean

**Fig. 3:**
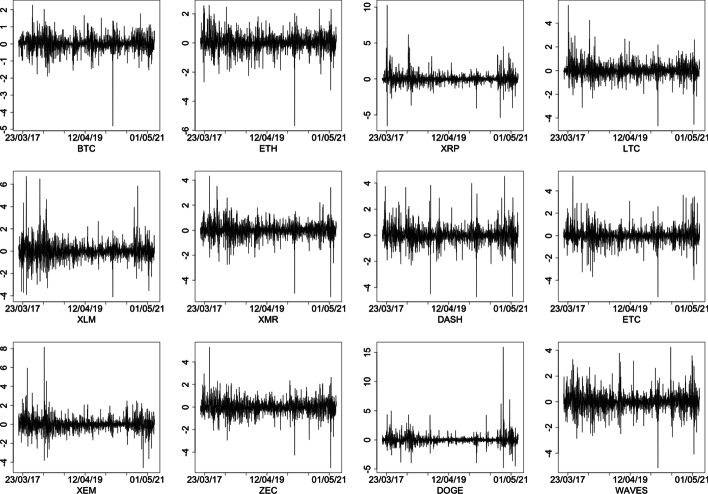
Dynamics of the 12 CCs’ log return considered

**Fig. 4:**
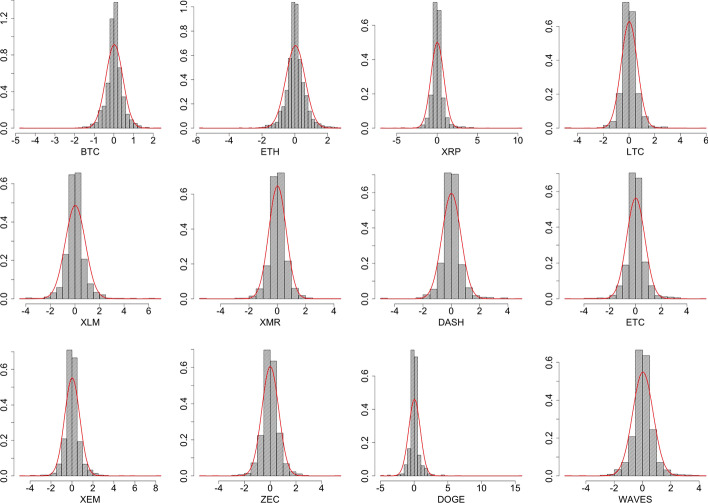
Histograms of the 12 CCs’ log return considered

The dynamics of $\vert r_{t}-\mu \vert$ are reported in Fig. 2, where $\mu$ is the mean of $r_{t}$, and show that the variances of the CCs’ log return series cannot be considered constant over time, whereas (investigated but not shown) the auto-correlations and cross-correlations for different lags in the CCs’ log return series can be considered—except for lag 0—zero with good approximation.

**Table 1:** Sample statistics of the 12 CCs’ log return considered

	Median	Interq. range	Skewness	Exc. Kurtosis	Min	Max
BTC	0.0025	0.0378	0.0254	1.9334	− 0.4799	0.2275
ETH	0.0016	0.0541	0.0959	1.5594	− 0.5656	0.2579
XRP	− 0.0000	0.0119	− 0.0342	4.9676	− 0.2288	0.2596
LTC	− 0.0002	0.0563	0.0110	1.2862	− 0.4543	0.4693
XLM	0.0000	0.0053	− 0.0413	6.7646	− 0.1243	0.1917
XMR	0.0022	0.0557	0.0013	1.5471	− 0.5301	0.4241
DASH	0.0001	0.0565	0.0061	1.7152	− 0.4679	0.4523
ETC	0.0003	0.0495	0.0395	2.0191	− 0.4572	0.4971
XEM	− 0.0001	0.0047	− 0.0306	8.4952	− 0.1844	0.2659
ZEC	− 0.0013	0.0658	0.0772	0.9853	− 0.5364	0.5295
DOGE	− 0.0000	0.0001	− 0.0673	114.0190	− 0.1033	0.1439
WAVES	0.0004	0.0447	0.0500	2.4293	− 0.3916	0.3377

We consider the model discussed in Eq. (1) to describe the evolution of the selected 12 CCs. First, we estimate the precision matrix for several values of the robustness parameter, namely, for $\gamma =0,0.01, 0.02, 0.05 \ \text{ and }\ 0.1$, and several bandwidth values, parameter, namely for $h=20, 50, 100 \ \text{ and }\ \infty$. We attempt different values of $\gamma$, and choose those that provide significantly different estimates. Therefore, we do not consider values $\gamma > 0.1$ because the estimates do not change significantly.

We stress that the estimation windows around the reference time are larger than the bandwidth; however, significant weights are given only to observations inside the bandwidth.

### Results

**Fig. 5:**
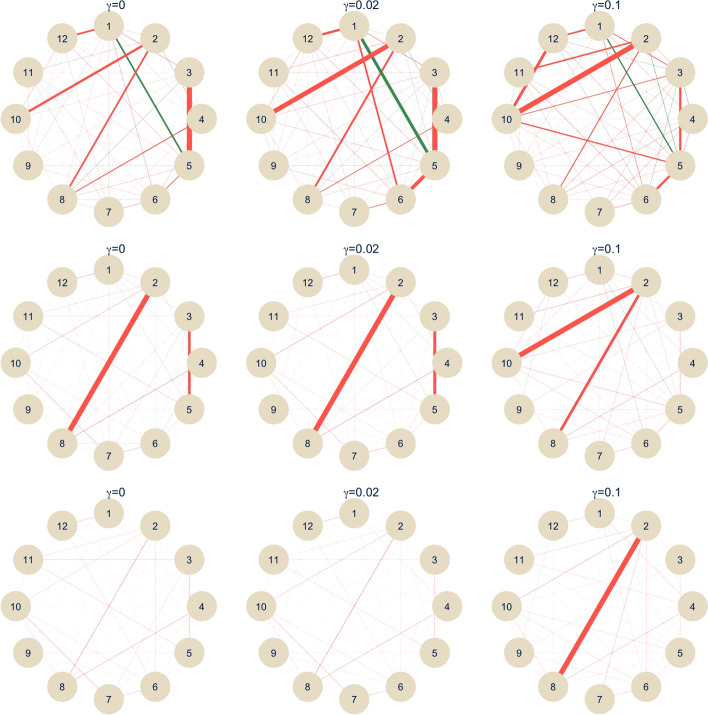
Conditional correlations around June 30 2017, using the bandwidth parameter $h=20$ (first row), $h=50$ (second row) and $h=100$ (third row). The parameter $\gamma$ refers to the robustness parameter: $\gamma =0$ means no robust approach

**Fig. 6:**
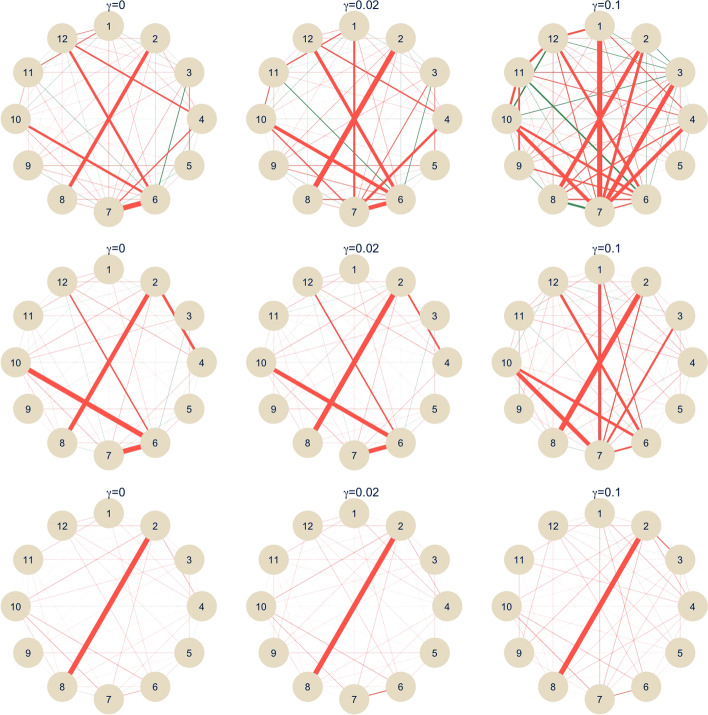
Conditional correlations around January 18 2018, using the bandwidth parameter $h=20$ (first row), $h=50$ (second row) and $h=100$ (third row). The parameter $\gamma$ refers to the robustness parameter: $\gamma =0$ means no robust approach

**Fig. 7:**
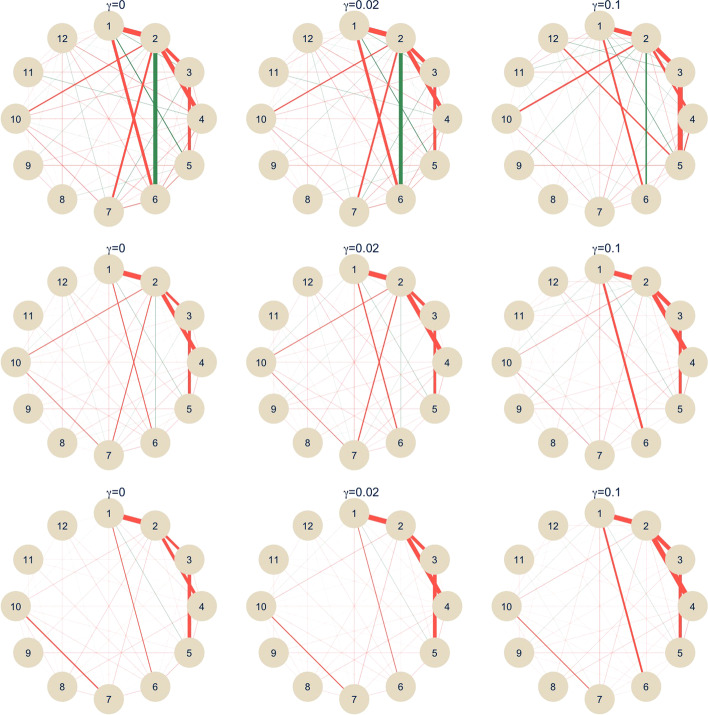
Conditional correlations around September 8 2019, using the bandwidth parameter $h=20$ (first row), $h=50$ (second row) and $h=100$ (third row). The parameter $\gamma$ refers to the robustness parameter: $\gamma =0$ means no robust approach

**Fig. 8:**
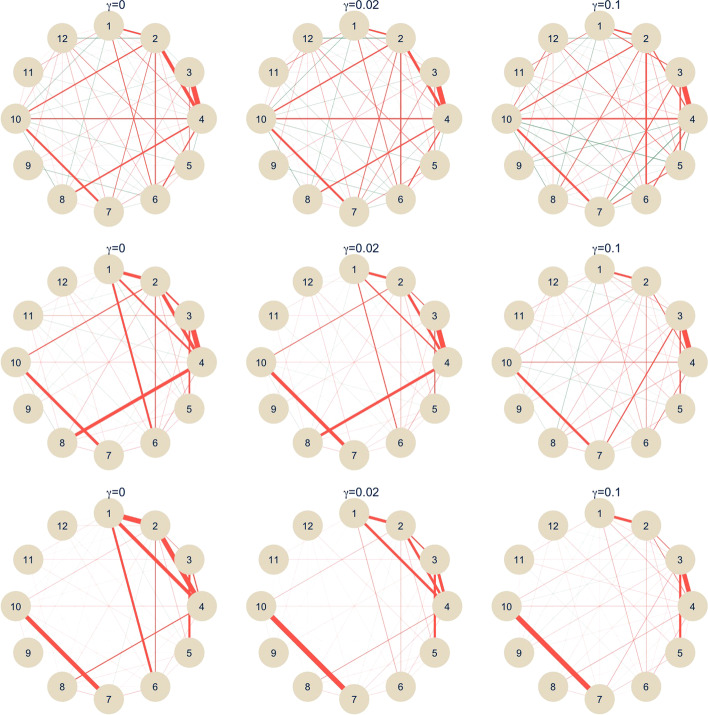
Conditional correlations around May 15, 2020, using the bandwidth parameter $h=20$ (first row), $h=50$ (second row) and $h=100$ (third row). The parameter $\gamma$ refers to the robustness parameter: $\gamma =0$ means no robust approach

**Fig. 9:**
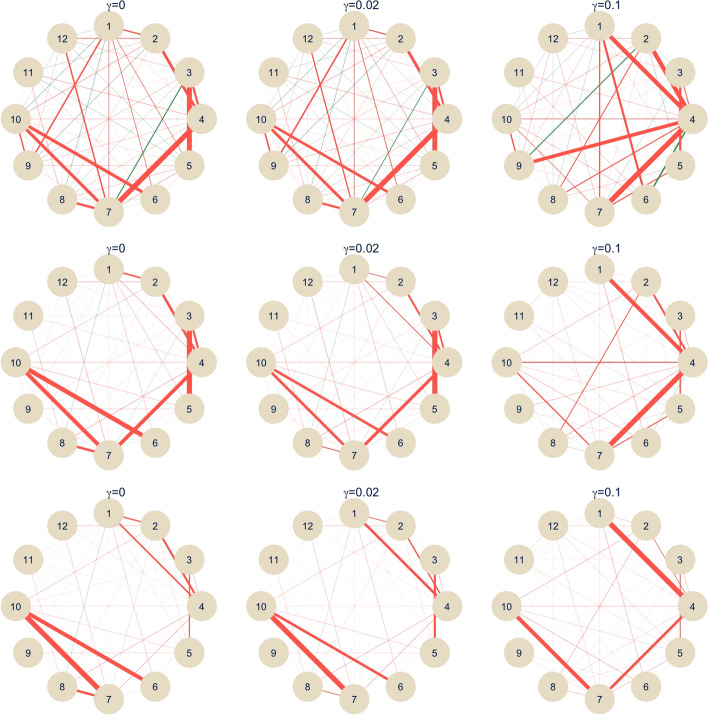
Conditional correlations around April 30 2021, using the bandwidth parameter $h=20$ (first row), $h=50$ (second row) and $h=100$ (third row). The parameter $\gamma$ refers to the robustness parameter: $\gamma =0$ means no robust approach

Precision matrices are used to construct Gaussian graphical models, which are statistical models describing relationships among variables in the form of graphs. Therefore, the estimated precision matrices are presented in “Conditional correlation graphs” section in the form of a graph. In particular, we show the five local estimates obtained considering a neighbor of the following dates: June 30, 2017, January 16, 2018, September 8, 2019, May 15, 2020, and April 30, 2021. The first date corresponds to a period in which investors started to observe the CCs market with more interest, the second date is immediately after the huge increase in Bitcoin—that is when the interest toward CCs was very high, and the third date corresponds to a period in which the interest toward this market was still high but not characterized by dramatic events. The last two dates correspond to the outbreak of the pandemic and to the second increase in the value of Bitcoin in April 2021, when its value reached 53,000 dollars. Figures 5, 6, 7, 8 and 9 shows that, in general, the smaller the value of the bandwidth of the kernel, the higher the interconnection between cryptocurrencies. This phenomenon results from the significantly not constant dependencies among the cryptocurrencies that, not being regulated by any institution and having no fundamentals from which to extract a price (Baek and Elbeck 2015; Huang et al. 2019) have wild variations that depend only on the whims of the market. With a large bandwidth, the time-varying dependency is mediated, resulting in a smaller interconnection. This evidence is the first of the importance of using a time-varying approach. Moreover, especially in Figs. 6 and 9, it is possible to observe high interconnection between cryptocurrency when the $\gamma$ parameter is high. These two figures are both associated with periods in which CCs are characterized by high growth and high volatility, and in the underlying process, CCs turn out to be conditionally dependent; however, the dependencies are hidden by extreme noisy events. Instead, Figs. 7 and 8 show how extreme events can distort the amplitude of the dependence between cryptocurrencies.

**Fig. 10:**
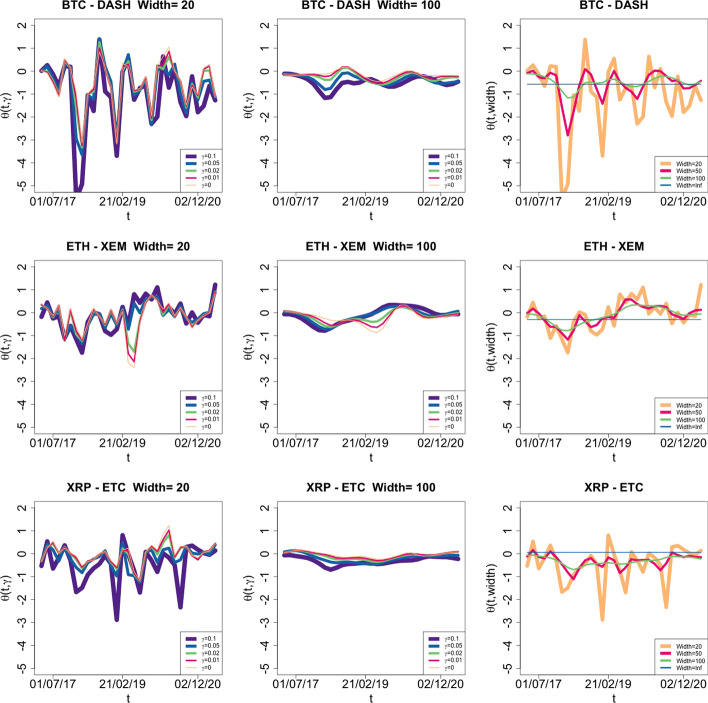
The plots show the dynamics of the conditional correlation between the 2 CCs reported in the title. In the first two column the value of the bandwidth is fixed at $h=20$ and $h=100$ respectively and each line in the plot corresponds to a parameter $\gamma$ as detailed in the legend. In the third column the value of gamma is fixed at $\gamma =0.1$ and each line in the plot corresponds to a bandwidth parameter *h* as detailed in the legend

What we have just said can be further detailed by observing Fig. 10 in “Conditional correlations dynamics” section, where we reported some conditional correlation dynamics, that is, the evolution of some elements of the precision matrix. Indeed, it turns out that, for a small value of *h* (first column), conditional correlations show a significant differences at varying $\gamma$, meaning that extreme events hide the underlying conditional correlation dynamics. Worth noting is that discrepancies among different estimates can be significant—more than one hundred percent. For high values of *h* (second column), the large bandwidth averaged the genuine time-dependent dynamics of the process, similar to what was observed from the synthetic stochastic model in “Time-varying estimation” section; however, differences in the estimation of the conditional correlation at varying $\gamma$ are still evident. Although the analysis is carried out on the 12 CCs, here we report only three examples of conditional correlations between CC pairs as an example of the type of analysis and the results that can be achieved using the proposed methodology.

**Fig. 11:**
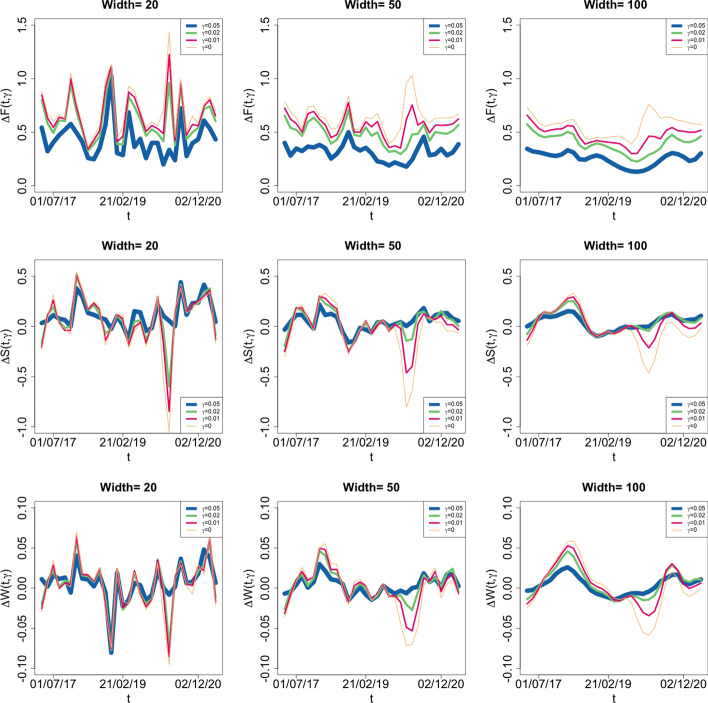
The plots show the dynamics of the measures defined in “Results” section. Each panel corresponds to a bandwidth *h*, in particular $h=20, 50, 100$ from left to right respectively. Each line in the plot corresponds to a measure, as reported on y-label

In Fig. 11 in “Quantitative measures” section, we consider a few indicators to quantify the discrepancies among the estimated precision matrices. The first indicator is the normalized Frobenius norm of the differences between the estimated precision matrix obtained for $\gamma =0.1$, for which we expect more accurate estimates and the one obtained for $\gamma =0,0.01, 0.02, 0.05$, that is

$$\begin{aligned} \varDelta F\left( t,\gamma \right) = \frac{\sqrt{\sum _{i,j}{\left( \varvec{\hat{\varTheta }}\left( t,0.1\right) -\varvec{\hat{\varTheta }}\left( t,\gamma \right) \right) ^{2}_{i,j}}}}{\sqrt{\sum _{i,j}{\left( \varvec{\hat{\varTheta }}\left( t,0.1\right) \right) ^{2}_{i,j}}}}. \end{aligned}$$

The computation of $\varDelta F\left( t,\gamma \right)$ is reported in the first row of Fig. 11, which shows that, even if this measure is an average quantity, the relative differences associated with the different values of $\gamma$ are remarkable for both small and large bandwidth values, touching one hundred percent for the smallest bandwidth.

Of course, the discrepancies among different estimates affect the computation of financial quantities. To measure such an impact, we also consider the following generally used quantities [see Billio et al. (2012) for more details]:

- the sum of the elements of the precision matrix, that is
  $$\begin{aligned} s\left( t, \gamma \right) =\sum _{i,j}\left| {\varvec{\hat{\varTheta }}\left( t, \gamma \right) _{i,j}}\right| , \end{aligned}$$
  gives a measure of interconnectedness of the entire system;
- let $\lambda _{k}\left( t,\gamma \right)$ for $k=1,\dots ,d$ be the eigenvalues of the correlation matrix $\hat{\varvec{\varSigma }}\left( t,\gamma \right)$ and $\nu \in \left\{ 1,\dots , d\right\}$, then
  $$\begin{aligned} w\left( t, \gamma \right) =\frac{\sum _{k=1}^{\nu }{\lambda _{k}\left( t,\gamma \right) }}{\sum _{k=1}^{d}{\lambda _{k}\left( t,\gamma \right) }} \end{aligned}$$
  is a measure of interconnectedness. Indeed, the numerator is the risk associated with the first $\nu$ principal components, whereas the denominator is the total risk of the system.

As previously done, we considered the relative differences of these measures using as reference the estimated precision matrix obtained for $\gamma =0.1$, for which we expect more accurate estimates, that is $\varDelta S\left( t,\gamma \right) = \frac{s\left( t, 0.1\right) -s\left( t, \gamma \right) }{s\left( t, 0.1\right) }$ and $\varDelta W\left( t, \gamma \right) = \frac{w\left( t, 0.1\right) - w\left( t,\gamma \right) }{w\left( t, 0.1\right) }$ for $\gamma =0,0.01, 0.02, 0.05$, to better highlight the effect of different estimations on the financial quantities of interest. We report the results in the second and third rows of Fig. 11, which show that both measures present significant differences by varying parameter $\gamma$. Such differences can also be observed when varying the bandwidth parameter, even if, as previously noted, at a small bandwidth ($h=20$), the differences are greater than those for the large bandwidth ($h=100$) because of the usual average effect that emerges in the last case.

Considering the three quantities reported in Fig. 11, the absolute value gradually decreases from $\varDelta F$ (first row), $\varDelta S$ (second row), and $\varDelta W$ (third row). This result is expected. In fact, $\varDelta F$, measuring the sum of the differences between the elements of the precision matrices, is an accurate measure of the discrepancies between the estimates obtained at varying $\gamma$, whereas $\varDelta S$, measuring the differences between the overall connections in the CC market (obtained at varying $\gamma$) can be considered a difference between composite variables that usually fluctuate less than the individual variables of which it is composed. Lastly, $\varDelta W$, measuring the relative differences in the percentages of variance explained by the first $\nu$ eigenvalues of the correlation matrix, is an extensive quantity least affected by different estimates obtained at varying $\gamma$.

## Discussion

The results shown in the previous section highlighted that time dependency is a relevant feature of the cryptocurrency market, that is, non-constant conditional dependencies among different cryptocurrencies, and different levels of contamination given by extreme events over time. We found further confirmation of our findings in recently published studies. For instance, Nie (2020) analyzed the correlation dynamics using a dimensionality reduction method. His analysis shows that the correlation dynamics experience drastic changes in relation to periods characterized by large market fluctuations. This finding is consistent with our observations. Indeed, the dynamics of conditional correlations show drastic changes when considering a small bandwidth and, thus, when having a short-term outlook. The changes in the dynamics of the conditional correlations decrease when a wider bandwidth is considered and, thus, when having a long-term outlook. Nie (2022) used a network method to identify critical events in the correlation dynamics of the CCs. He found that the network structure is easily broken near critical events, namely, periods characterized by large market fluctuations. After these events, the network structure returns to stability. We observed something similar; indeed, the graphs we obtained using conditional correlations change their structure in correspondence of large market fluctuations. These changes are reduced when considering a wider bandwidth, that is, from a long-term perspective. In Shams (2020), the authors investigated the structure of CC returns and found persistence among CCs’ sharing similar features. The author introduced a connectivity measure that captures strong exchange-specific commonalities in CCs’ investors’ demand, which spills over to other exchanges. This spillover could explain the drastic changes in the conditional correlations we observe when having a short-term outlook. Indeed, the author suggests that demand from users and developers might correlate with that of investors and speculators, translating into an amplified effect on prices and explaining that CCs are prone to wild price movements and bubbles. In Guo et al. (2021), the authors investigated the market segmentation problem and information propagation mechanism in the CCs market by constructing a time-varying network of CCs that combines return cross-predictability and technological similarities. The network based on return cross-predictability is obtained using an adaptive Lasso regression technique, which is very similar to our approach in which the elements of the precision matrix can also be obtained as coefficients of a linear regression model. However, the manner in which they addressed the time dependency is different from ours because they considered rolling windows. All of these results unveil the complexity of the cryptocurrency market and any type of investment strategy, such as high frequency trading or long-term hedging techniques, must be carefully planned using the right quantitative tools. Of course, other indicators could be considered to test the interconnectedness of the CCs market or to assess the performances of a portfolio containing CCs; however, it is out of the scope of this paper, which has the aim of providing a flexible and robust estimation methodology able to manage different levels of data contamination and time-varying parameters at different time scales.

Finally, some considerations on the choice of the parameters $\gamma$ and *h* are in order. Figures 10 and 11 provide insights into the influence of *h* and $\gamma$ on parameter estimations. In general, the choice of *h* depends on the type of analysis that is intended to be carried out, such as long-term portfolio management that needs a large estimation window, whereas high frequency analysis (e.g., to grasp time-varying correlations) needs a short estimation window. In the CCs market, characterized by highly non-stationary signals, parameter *h* acts as a smoother over the time domain; namely, a higher *h* results in smoother dynamics of the parameter estimates. In other words, small values of parameter *h*, which identifies the estimation window size, favor a precise local estimation of the model parameters at the expense of the smoothness of their dynamics. Instead, parameter $\gamma$ provides significant differences in parameter estimations when the data are highly non-Gaussian. Indeed, Fig. 11 and, in particular, the plots showing the dynamics of the $\varDelta S\left( t,\gamma \right)$ measure for different $\gamma$ values indicate that such dynamics remarkably diverge at a few points corresponding to the first significant increase in Bitcoin in December 2017, when its value reached 16,000 dollars, to the outbreak of the Covid-19 pandemic and to the second significant increase in Bitcoin in April 2021, when the value reached 53,000 dollars. Therefore, *h* and $\gamma$ must be independently set according to the need for local estimations and different data contamination levels. To conclude, we highlight the fact that, for what was just discussed, no unique optimal choice of the hyperparameters exists, and it is strictly related to the analysis of interest.

## Conclusions

The main motivation for this work is the introduction of a local and robust estimator of precision matrices able to handle both extreme events and time-varying parameters that typically affect financial time series. In particular, the proposed estimator, obtained by using a kernel over the time domain and replacing the Kullbach–Leibler divergence with a robust divergence, namely, the $\gamma$ divergence, is applied to estimate time-varying precision matrices of the log return of a representative subset of the cryptocurrency market. The proposed method is remarkably suited for the considered application because the cryptocurrency market is characterized by a peculiar time-dependent and very turbulent nature because the major movements on it are the result of speculators who are free to act in a market not regulated by any authority. Moreover, the application is particularly relevant for both the increasing interest in the cryptocurrency market—witnessed by the growing literature and investment in CCs—and the attention that the precision matrix gained in the financial context, given its various and useful applications, from portfolio optimization to systemic risk analysis.

When the financial time series show cyclic or trend components, a simple Gaussian process cannot be considered a reliable model. In such cases, the model considered in this work can be easily extended by considering regression terms, and the proposed estimator could be used with few modifications to the algorithm.

Moreover, in high-dimensional settings—when the number of features is higher than the number of observations—sparsity can be introduced in the precision matrix’s structure by adding a penalty to the local estimated $\gamma$-divergence.

In addition, clustering algorithms can be used to classify time-varying precision matrices to detect turbulent periods and minimize investor risk (Kou et al. 2014, 2021a, b; Li et al. 2021).

Finally, the use of a kernel estimator together with an M-type estimator has been introduced by Cai and Ould-Saïd (2003), in which the authors proposed a robust version of a local linear regression smoother for stationary time series. They also extended to the case of non-i.i.d. random variables of the asymptotic theory developed by Fan and Jiang (2000), in which the authors studied local robust techniques in the i.i.d. case. We are working on adapting the asymptotic theory developed in Cai and Ould-Saïd (2003) to our estimator. The technicalities and mathematical proofs, which are out of the scope of this paper, will be published in a separate paper.

## Data Availability

The data is public because it comes from a data base company called CoinMarketCap.

## Appendix 1: Figures

### Cryptocurrencies log return

See Figs. 3 and 4.

### Conditional correlation graphs

See Figs. 5, 6, 7, 8 and 9.

### Conditional correlations dynamics

See Fig. 10.

### Quantitative measures

See Fig. 11.

## Appendix 2: Algorithm

Given the model in Eq. (1), the parameters being estimated through minimisation problem in Eq. (7) correspond to elements in the precision matrix $\varvec{\varTheta }_{t}$, for $0\le t\le T$. Therefore, let $\varvec{\theta }_t=\mathrm{vec}\left( \varvec{\varTheta }_t\right) ^\prime$ be the vector of parameters at time *t*, and $\widehat{\varvec{\theta }}_t^{(j)}$ be an estimate of $\varvec{\theta }_{t}$ at the *j*th iteration. Let us denote

$$\begin{aligned} z_u^{(j)}&=\frac{w_{ut}f\left( \varvec{r}_u,\widehat{\varvec{\theta }}_t^{(j)}\right) ^\gamma }{\sum _{k=1}^{T}w_{kt}f\left( \varvec{r}_k,\widehat{\varvec{\theta }}_t^{(j)}\right) ^\gamma },\\ x_u^{(j)}&=\sum _{k=1}^{T}w_{kt}f\left( \varvec{r}_k,\widehat{\varvec{\theta }}_t^{(j)}\right) ^\gamma \frac{w_{ut}f\left( \varvec{r}_u,\varvec{\theta }_t\right) ^\gamma }{w_{ut}f\left( \varvec{r}_u,\widehat{\varvec{\theta }}_t^{(j)}\right) ^\gamma }, \end{aligned}$$

so that $z_u^{(j)} x_u^{(j)}=w_{ut}f\left( \varvec{r}_u,\varvec{\theta }_t\right) ^\gamma$. Then, using the Jensen’s inequality to the convex function $y=-\log \left( x\right)$, we get

8 $$\begin{aligned} - \frac{1}{\gamma }\ln \left\{ {\sum\limits_{{u = 1}}^{T} {w_{{ut}} } f\left( {\varvec{r}_{u} ,\varvec{\theta }_{t} } \right)^{\gamma } } \right\} & = - \frac{1}{\gamma }\ln \left\{ {\sum\limits_{{u = 1}}^{T} {z_{u}^{{(j)}} } x_{u}^{{(j)}} } \right\} \le - \frac{1}{\gamma }\sum\limits_{{u = 1}}^{T} {z_{u}^{{(j)}} } \log x_{u}^{{(j)}} \\ & = - \frac{1}{\gamma }\sum\limits_{{u = 1}}^{T} {z_{u}^{{(j)}} } \log \left[ {\sum\limits_{{k = 1}}^{T} {w_{{kt}} } f\left( {\varvec{r}_{k} ,\varvec{\hat{\theta }}_{t}^{{(j)}} } \right)^{\gamma } \frac{{w_{{ut}} f\left( {\varvec{r}_{u} ,\varvec{\theta }_{t} } \right)^{\gamma } }}{{w_{{ut}} f\left( {\varvec{r}_{u} ,\user2{\hat{\theta }}_{t} ^{{(j)}} } \right)^{\gamma } }}} \right] \\ & = - \frac{1}{\gamma }\sum\limits_{{u = 1}}^{T} {z_{u}^{{(j)}} } \log \left[ {\frac{{w_{{ut}} f\left( {\varvec{r}_{u} ,\varvec{\theta }_{t} } \right)^{\gamma } }}{{z_{u}^{{(j)}} }}} \right] \\ & = - \frac{1}{\gamma }\sum\limits_{{u = 1}}^{T} {z_{u}^{{(j)}} } \log \left[ {w_{{ut}} f\left( {\varvec{r}_{u} ,\varvec{\theta }_{t} } \right)^{\gamma } } \right] + \frac{1}{\gamma }\sum\limits_{{u = 1}}^{T} {z_{u}^{{(j)}} } \log z_{u}^{{(j)}} = :\tilde{\ell }_{{1,t}} \left( {\varvec{r},\varvec{\theta }_{t} |\varvec{\hat{\theta }}_{t} ^{{(j)}} } \right). \\ \end{aligned}$$

Moreover

9 $$\begin{aligned} \tilde{\ell }_{{1,t}} \left( {\varvec{r},\varvec{\hat{\theta }}_{t}^{{(j)}} |\varvec{\hat{\theta }}_{t}^{{(j)}} } \right) & = - \frac{1}{\gamma }\sum\limits_{{u = 1}}^{T} {z_{u}^{{(j)}} } \log \left[ {\sum\limits_{{k = 1}}^{T} {w_{{kt}} } f\left( {\varvec{r}_{k} \varvec{,\hat{\theta }}_{t}^{{(j)}} } \right)^{\gamma } \frac{{w_{{ut}} f\left( {\varvec{r}_{u} \varvec{,\hat{\theta }}_{t}^{{(j)}} } \right)^{\gamma } }}{{w_{{ut}} f\left( {\varvec{r}_{u} \varvec{,\hat{\theta }}_{t}^{{(j)}} } \right)^{\gamma } }}} \right] \\ & = - \frac{1}{\gamma }\sum\limits_{{u = 1}}^{T} {\frac{{w_{{ut}} f\left( {\varvec{r}_{u} \varvec{,\hat{\theta }}_{t}^{{(j)}} } \right)^{\gamma } }}{{\sum\limits_{{k = 1}}^{T} {w_{{kt}} } f\left( {\varvec{r}_{k} \varvec{,\hat{\theta }}_{t}^{{(j)}} } \right)^{\gamma } }}} \log \left[ {\sum\limits_{{k = 1}}^{T} {w_{{kt}} } f\left( {\varvec{r}_{k} \varvec{,\hat{\theta }}_{t}^{{(j)}} } \right)^{\gamma } } \right] \\ & = - \frac{1}{\gamma }\log \left[ {\sum\limits_{{k = 1}}^{T} {w_{{kt}} } f\left( {\varvec{r}_{k} \varvec{,\hat{\theta }}_{t}^{{(j)}} } \right)^{\gamma } } \right] = \ell _{{1,t}} \left( {\varvec{r,\hat{\theta }}_{t}^{{(j)}} } \right) \\ \end{aligned}$$

Therefore by Eqs. (8) and (9) it holds

$$\tilde{\ell }_{{1,t}} \left( {\varvec{r},\varvec{\theta }_{t} |\varvec{\hat{\theta }}_{t}^{{(j)}} } \right) \ge \ell _{{1,t}} \left( {\varvec{r},\varvec{\theta }_{t} } \right)\quad {\text{ and }}\quad \tilde{\ell }_{{1,t}} \left( {\varvec{r},|\varvec{\hat{\theta }}_{t}^{{(j)}} |\varvec{\hat{\theta }}_{t}^{{(j)}} } \right) = \ell _{{1,t}} \left( {\varvec{r},\varvec{\hat{\theta }}_{t}^{{(j)}} ,} \right),$$

that are the two properties characterising a majorisation function, therefore allowing to use an MM–algorithm in order to solve the minimisation problem in Eq. (7).

At iteration $j+1$ the following step have to be implemented:

- Majorize–Step: compute
  $$\tilde{\ell }_{{\gamma ,t}} \left( {\varvec{r},\varvec{\theta }_{t} |\varvec{\hat{\theta }}_{t}^{{(j)}} } \right) = \tilde{\ell }_{{1,t}} \left( {\varvec{r},\varvec{\theta }_{t} |\varvec{\hat{\theta }}_{t}^{{(j)}} } \right) + \ell _{2} \left( {\varvec{\theta }_{t} } \right).$$
- Minimisation–Step: solve
  $$\varvec{\hat{\theta }}_{t}^{{(j + 1)}} = \arg \min _{\varvec{\theta }} \tilde{\ell }_{{\gamma ,t}} \left( {\varvec{r},\varvec{\theta }_{t} |\varvec{\hat{\theta }}_{t}^{{(j)}} } \right).$$

In order to solve the minimisation step we use the results in the “Appendix 2” of Hirose et al. (2017) regarding the function $\ell _{2}\left( \varvec{\theta }_{t}\right)$. In particular, it holds

$$\begin{aligned} \ell _{2}\left( \varvec{\theta }_{t}\right) = \frac{1}{1+\gamma }\left( -\frac{\gamma p}{2}\log 2\pi +\frac{\gamma }{2}\log \vert \varvec{\varTheta }_{t}\vert -\frac{p}{2}\log \left( 1+\gamma \right) \right) \end{aligned}$$

and

10 $$\tilde{\ell }_{{1,t}} \left( {\user2{r},\varvec{\theta }_{t} |\varvec{\hat{\theta }}_{t}^{{(j)}} } \right) = \frac{1}{2}{\text{ tr }}\left( {\varvec{\varTheta }_{t} \varvec{\hat{S}}_{t}^{{(j)}} } \right) + \frac{1}{2}\log |{\text{ }}\varvec{\varTheta }_{t} | + c.$$

where $\left( {\varvec2{\hat{S}}_{t}^{{(j)}} } \right)_{{i,j}} = \sum\limits_{{s = 1}}^{T} {z_{s}^{{(j)}} } \varvec{r}_{{i,s}} \varvec{r}_{{j,s}}^{\prime }$ and *c* is a constant.

Therefore the following formula is obtained

$$\begin{aligned} \tilde{\ell }_{\gamma , t}\left( \varvec{r}, \varvec{\theta }_{t}\vert \varvec{\hat{\theta }}_{t}^{{(j + 1)}} \right)&=\frac{1}{2}\text{ tr }\left( {\varvec{\varTheta }_{t} \varvec{\hat{S}}_{t}^{{(j)}} } \right) -\frac{1}{2\left( 1+\gamma \right) }\log \vert \varvec{\varTheta }_{t}\vert +\tilde{c}. \end{aligned}$$

To find the update $\varvec{\theta }_t^{(j+1)}$ we need to solve the following minimisation problem

11 $$\begin{aligned} {\varvec{\hat{\theta }}_{t} ^{{(j + 1)}} } &=\arg \min _{\varvec{\theta }_t}\frac{1}{2}\text{ tr }\left( {\varvec{\varTheta }_{t} \varvec{\hat{S}}_{t}^{{(j)}} } \right) -\frac{1}{2\left( 1+\gamma \right) }\log \vert \varvec{\varTheta }_{t}\vert \end{aligned}$$

whose solution is carried out similarly to maximum–likelihood method.

It is interesting to notice that, if we want to introduce sparsity in the precision matrix, the detailed algorithm would work in the same way and the minimisation step would require to solve the following

$$\begin{aligned} {\varvec{\hat{\theta }}_{t} ^{{(j + 1)}} } &=\arg \min _{\varvec{\theta }_t}\frac{1}{2}\text{ tr }\left( {\varvec{\varTheta }_{t} \varvec{\hat{S}}_{t}^{{(j)}} } \right) -\frac{1}{2\left( 1+\gamma \right) }\log \vert \varvec{\varTheta }_{t}\vert +\lambda \vert \varvec{\varTheta }_{t}-\text{ diag }\left( \varvec{\varTheta }_{t}\right) \vert _{1} \end{aligned}$$

that can be solved using the graphical lasso algorithm, see Friedman et al. (2008).

## Abbreviations

CC: Cryptocurrency
